# Paeoniflorin in Paeoniaceae: Distribution, influencing factors, and biosynthesis

**DOI:** 10.3389/fpls.2022.980854

**Published:** 2022-09-02

**Authors:** Xiao-Xiao Zhang, Jia-Qi Zuo, Yi-Ting Wang, Hui-Yun Duan, Jun-Hui Yuan, Yong-Hong Hu

**Affiliations:** ^1^College of Landscape Architecture and Arts, Northwest A&F University, Yangling, Shaanxi, China; ^2^Shanghai Key Laboratory of Plant Functional Genomics and Resources, Shanghai Chenshan Botanical Garden, Shanghai, China

**Keywords:** Paeoniaceae, Paeoniflorin, influencing factors, biosynthesis, post-modification enzyme, Monoterpene

## Abstract

Paeoniflorin, a monoterpene glucoside, is increasingly used in the clinical treatment of many diseases because it has a variety of pharmacological activities. Besides, paeoniflorin has been considered the characteristic chemical constituent of Paeoniaceae plants since it was first reported in 1963. Therefore, how to better develop and utilize paeoniflorin in Paeoniaceae has always been a research hotspot. We reviewed the current knowledge on paeoniflorin in Paeoniaceae, with particular emphasis on its distribution and influencing factors. Moreover, the limited understanding of the biosynthesis pathway has restricted the production of paeoniflorin by synthetic biology. This review provides insights into the post-modification pathway of paeoniflorin biosynthesis and proposes directions for further analysis in the future.

## Introduction

Paeoniaceae is a single genus family, and *Paeonia* consists of 34 species, comprising shrubs and perennial herbs mainly distributed in temperate Eurasia, northwest Africa, and western North American (Hong, 2010, 2021). The shrubs, called tree peonies, belong to Section *Moutan* DC., and the perennials, called herbaceous peonies, are classified into Sect. *Paeonia* or Sect. *Onaepia* Lindley (Hong, 2011). China has the most concentrated distribution of Paeoniaceae plants, and it is also one of the centers of differentiation and development of existing Paeoniaceae primitive groups (Zhou et al., 2021). Since the Eastern Han Dynasty (25–220 A.D.), Paeoniaceae plants have been widely cultivated and used in China because of their special medicinal effects (Li et al., 2011). Currently, there are three traditional Chinese medicines in China, Mudanpi, Baishao, and Chishao, which are processed from the roots of Paeoniaceae plants (Chinese Pharmacopoeia Committee, 2020). These three medicines have the functions of clearing heat, cooling blood, promoting blood circulation, and removing blood stasis (Tan et al., 2020; Wu et al., 2020).

Although there are many active components in Paeoniaceae plants, paeoniflorin, a monoterpene bicyclic glycoside, is considered the most important medicinal ingredient (Xiu et al., 2021). Modern medical studies have shown that paeoniflorin has immunoregulatory, antidepressant, anti-arthritis, antithrombosis, anti-tumor, hepatoprotective, cerebral ischemic injury protective, and neuroprotective effects (Zhang et al., 2019a; Zhang, 2020). Moreover, paeoniflorin, found only in Paeoniaceae plants in nature, is considered the characteristic constituent of Paeoniaceae (Yu and Xiao, 1987). Therefore, numerous studies have focused on the distribution of paeoniflorin in Paeoniaceae to better develop and utilize these plants for medicinal purposes (He, 2010; Yang et al., 2020; Yan et al., 2021). As a secondary metabolite, the content of paeoniflorin is affected by many factors, including biological and abiotic factors (Chen et al., 2009; Feng et al., 2012; Shen et al., 2019; Zhang and Wei, 2020). The influence of these factors on the content of paeoniflorin has become a topic of research interest. Our research group has also carried out several studies on paeoniflorin in Paeoniaceae plants (Zhang et al., 2019b). Thus, it is necessary to systematically review this research and establish expectations for future studies.

Currently, paeoniflorin is mainly extracted from the roots of Paeoniaceae plants, but this has many disadvantages, such as low yield, difficulty in separation of extracts, and wastage of plant resources (Nie et al., 2005). Although paeoniflorin has been synthesized by chemical methods for a long time, it is not used in actual production because of its complicated process, high production cost, and contaminated production process (Corey and Wu, 1993; Hatakeyama et al., 1994). As an active natural product with important medicinal value, paeoniflorin is increasingly required for drug preparation; for this reason, it is important to realize its large-scale production economically. The biosynthesis of natural products is one of the most important research directions in synthetic biology. Compared with the two abovementioned methods, biosynthesis has many advantages, such as a single product, high yield, an environmentally friendly process, and no restriction of raw materials (Sun et al., 2017). However, the biosynthesis pathway of paeoniflorin has not been fully elucidated, especially the modification stage that determines its structural and functional specificity. There are few studies on the enzymes involved in this stage, which fundamentally limit the production of paeoniflorin by synthetic biology.

In this review, we summarized the distribution characteristics of paeoniflorin and the effects of different factors on paeoniflorin content in Paeoniaceae. More importantly, we predicted the post-modification pathway of paeoniflorin biosynthesis and analyzed the paeoniflorin biosynthesis pathway.

## Paeoniflorin in Paeoniaceae

### Characteristic constituents of Paeoniaceae

The secondary metabolites in Paeoniaceae are complex, with more than 300 chemical components identified, mainly including monoterpene glycosides, triterpenoids, flavonoids, tannins, phenolic acids, sugars, steroids, and volatile oils (He et al., 2010). Paeoniflorin was first isolated from the roots of *Paeonia albiflora* and named by Shibata et al. (1963), who pointed out that paeoniflorin was D-glucoside with a benzoylated C_10_-compound (C_10_H_14_O_5_). Further studies showed that paeoniflorin is a monoterpene glucoside whose basic skeleton is a pinane derivative (Shibata et al., 1964; Aimi et al., 1969; Kaneda et al., 1972). Subsequently, an increasing number of species belonging to Paeoniaceae have been reported to contain paeoniflorin in their roots (Egon and Cooper, 1969). Paeoniflorin has always only been found in Paeoniaceae plants (Yu and Xiao, 1987). Therefore, paeoniflorin was naturally considered to be the characteristic constituent of Paeoniaceae until 2008, when it was isolated from the water fern *Salvinia molesta* (Choudhary et al., 2008), which was the first report that paeoniflorin occurred in plants other than Paeoniaceae. In our previous study (Zhang et al., 2019b), we did not detect paeoniflorin in *S. molesta* by UPLC-MS, nor did we detect it in any of the 16 plants from different families. In addition to the roots of Paeoniaceae plants, other organs, including flowers, stems, leaves, fruits, seeds, and rhizomes, have also been investigated, and the results show that paeoniflorin is distributed throughout the plant. These results have thus confirmed that paeoniflorin is a characteristic constituent of Paeoniaceae without tissue specificity.

Although paeoniflorin occurs widely in the roots of species belonging to Paeoniaceae, its contents were quite different (Table 1; Supplementary Table 1). He et al. (1980) determined the paeoniflorin content in the roots of 11 species and varieties, and the content ranged from 0.09 to 10.72%. Yu and Xiao (1985, 1987) showed that the paeoniflorin content of 19 species and 6 varieties ranged from 0.40 to 4.36%, and a study by Jin et al. (1989) on 13 species and varieties found paeoniflorin contents ranging from 0.76 to 7.78%. Recently, our study included 16 species that exhibited a wide range of paeoniflorin content, from 0.22 to 5.12% (Zhang et al., 2019b). Many studies with a few samples (Han et al., 2008; Yang et al., 2020; Yan et al., 2021; Li et al., 2021a) that were not representative have shown that the paeoniflorin content in the roots of different species differs. In all the above studies, the roots varied by sampling time and location, and the detection methods of paeoniflorin were different; therefore, there is no agreement on which species have the highest and lowest paeoniflorin content. However, the consistent conclusion was that the paeoniflorin content of herbaceous peony roots was higher than that of tree peony roots. Furthermore, most studies have concluded that the paeoniflorin content in the roots of species collected from the wild habitat was higher than that in the roots of species collected from the cultivated habitat (Hu et al., 2000; Guo et al., 2002; Zhou et al., 2003; Wang et al., 2012).

**Table 1:** Paeoniflorin content in the roots of different species belonging to Paeoniaceae.

**Species**	**Method**	**Content (mg/g)**	**References**
*Paeonia suffruticosa*	TLC	9.0–25.0^*^	He et al., 1980; Yu and Xiao, 1987
*P. suffruticosa*	HPLC	14.1–25.6^*^	Guo et al., 2002
*Paeonia ostii*	HPLC	17.4^*^	Guo et al., 2002
*P. ostii*	HPLC-MS	5.8–7.6	Zhang et al., 2019b; Yan et al., 2021
*Paeonia qiui*	HPLC-MS	14.0–24.2	Zhang et al., 2019b; Yan et al., 2021
*Paeonia rockii*	HPLC	13.7–46.8^*^	Guo et al., 2002
*P. rockii*	HPLC-MS	6.0–8.7	Zhang et al., 2019b; Yan et al., 2021
*Paeonia jishanensis*	HPLC-MS	17.2–19.6	Zhang et al., 2019b; Yan et al., 2021
*Paeonia decomposita*	HPLC	43.4–48.2^*^	Guo et al., 2002
*P. decomposita*	HPLC-MS	12.3–14.0	Zhang et al., 2019b; Yan et al., 2021
*Paeonia rotundiloba*	HPLC-MS	9.8	Yan et al., 2021
*Paeonia szechuanica*	TLC	7.9–18.8^*^	Yu and Xiao, 1987
*Paeonia delavayi*	TLC	7.8–19.3^*^	He et al., 1980; Yu and Xiao, 1987
*P. delavayi*	HPLC-MS	1.9–16.2	Zhang et al., 2019b; Yan et al., 2021
*P. delavayi* var. *lutea*	TLC	14.2–25.2^*^	He et al., 1980; Yu and Xiao, 1987
*P. delavayi* var. *lutea*	RT-HPLC	7.6–18.2^*^	Jin et al., 1989
*Paeonia ludlowii*	HPLC-MS	0.9–2.3	Zhang et al., 2019b; Yan et al., 2021
*Paeonia lactiflora*	TLC	28.1–107.2^*^	He et al., 1980; Yu and Xiao, 1987
*P. lactiflora*	RT-HPLC	23.3–75.5^*^	Jin et al., 1989
*P. lactiflora*	HPLC-MS	22.2–92.3	Zhang et al., 2019b; Yang et al., 2020; Yan et al., 2021
*P. lactiflora* var. *trichocarpa*	TLC	49.6–57.0^*^	He et al., 1980
*P. lactiflora* var. *trichocarpa*	RT-HPLC	64.5–77.8^*^	Jin et al., 1989
*Paeonia veitchii*	TLC	13.4–57.6^*^	He et al., 1980; Yu and Xiao, 1987
*P. veitchii*	RT-HPLC	41.3–52.1^*^	Jin et al., 1989
*P. veitchii*	HPLC-MS	12.1–90.6	Yuan et al., 2020; Yang et al., 2020
*P. veitchii* var. *uniflora*	TLC	34.7^*^	Yu and Xiao, 1987
*P. veitchii* var. *woodwardii*	TLC	9.6^*^	Yu and Xiao, 1987
*P. veitchii* var. *woodwardii*	RT-HPLC	33.8*	Jin et al., 1989
*Paeonia obovata*	TLC	2.7–24.6^*^	He et al., 1980; Yu and Xiao, 1987
*P. obovata*	RT-HPLC	14.7^*^	Jin et al., 1989
*P. obovata*	HPLC-MS	0.5–33.5	Zhang et al., 2019b; Yang et al., 2020; Yan et al., 2021
*P. obovata* var. *willmottiae*	TLC	0.9–25.4^*^	He et al., 1980
*P. obovata* var. *willmottiae*	RT-HPLC	38.2^*^	Jin et al., 1989
*P. obovata* ssp*. willmottiae*	HPLC-MS	6.9–21.6	Zhang et al., 2019b; Yang et al., 2020; Yan et al., 2021
*Paeonia emodi*	TLC	5.6^*^	Yu and Xiao, 1987
*P. emodi*	HPLC-MS	24.5–51.7	Zhang et al., 2019b; Yang et al., 2020; Yan et al., 2021
*Paeonia sterniana*	HPLC-MS	24.7–53.3	Zhang et al., 2019b; Yang et al., 2020; Yan et al., 2021
*Paeonia intermedia*	HPLC-MS	3.1–18.9	Zhang et al., 2019b; Yang et al., 2020; Yan et al., 2021
*Paeonia mairei*	TLC	26.6–27.2^*^	He et al., 1980; Yu and Xiao, 1987
*P. mairei*	RT-HPLC	14.0–30.7^*^	Jin et al., 1989
*P. mairei*	HPLC-MS	14.5–29.5	Zhang et al., 2019b; Yang et al., 2020; Yan et al., 2021
*Paeonia anomala*	TLC	13.0^*^	Yu and Xiao, 1987
*P. anomala*	RT-HPLC	26.2^*^	Jin et al., 1989
*P. anomala*	HPLC-MS	6.9–64.7	Zhang et al., 2019b; Yang et al., 2020; Yan et al., 2021
*P. anomala* var. *intermedia*	TLC	13.9–24.0^*^	He et al., 1980; Yu and Xiao, 1987
*P. anomala* var. *intermedia*	RT-HPLC	22.1^*^	Jin et al., 1989
*Paeonia sinkiangensis*	TLC	13.3–14.8^*^	He et al., 1980; Yu and Xiao, 1987
*P. sinkiangensis*	RT-HPLC	29.5^*^	Jin et al., 1989
*Paeonia japonica*	RT-HPLC	11.1^*^	Jin et al., 1989
*Paeonia officinalis*	TLC	16.1–25.7^*^	Yu and Xiao, 1987
*Paeonia decora*	TLC	17.8–21.6^*^	Yu and Xiao, 1987
*Paeonia tenuifolia*	TLC	23.1–42.5^*^	Yu and Xiao, 1987
*Paeonia bakeri*	TLC	16.2^*^	Yu and Xiao, 1987
*Paeonia paradoxa*	TLC	6.8^*^	Yu and Xiao, 1987
*Paeonia humilis* var. *villosa*	TLC	14.4^*^	Yu and Xiao, 1987
*Paeonia banatica*	TLC	14^*^	Yu and Xiao, 1987
*Paeonia corallina*	TLC	8.3^*^	Yu and Xiao, 1987
*P. coriacea* var. *atlantica*	TLC	4^*^	Yu and Xiao, 1987

* The content in the original study was expressed as the percentage.

### Factors influencing paeoniflorin content in Paeoniaceae

#### Plant ages

Some studies have suggested that older *Paeonia lactiflora* plants have a higher paeoniflorin content in their roots (Huang et al., 2000; Zhang, 2000; Jian et al., 2010). However, if the roots are more than 5 years old, the paeoniflorin content decreases (Jian et al., 2010; Kang, 2011). Other studies have reported contrasting results, that is, the paeoniflorin content in the roots is negatively correlated with plant age (Xue et al., 1992; Li et al., 2008; Yan et al., 2019). The reason for this may be that the starch in the roots increases with the growth of the plant, and paeoniflorin decomposes *in vivo* (Xue et al., 1992). Our studies have shown that there is no significant correlation between paeoniflorin content and plant age in either the roots of *Paeonia ostii* or in the roots of *P. lactiflora* (Zhang et al., 2019b). In conclusion, plant ages affect paeoniflorin content in the roots (Supplementary Table 2), but there is no unified conclusion on this effect.

#### Development stages

Paeoniaceae are perennial herbs or woody shrubs, with flower buds and leaf buds sprouting in early spring and falling into dormancy in autumn. Their development stages can be divided into budding, leaf unfolding, buds, flowering, fruiting, and withering (Li et al., 2011). Numerous studies have confirmed that the paeoniflorin contents in the roots at different developmental stages are significantly different (Supplementary Table 3). With the development of plants, the paeoniflorin content of the roots first increased and then decreased. The highest content was usually observed in spring (budding or flowering stages; He et al., 1980; Yu and Xiao, 1985; Jin et al., 1991, 2010; Huang et al., 2000; Fu et al., 2020). It seems best to harvest roots in spring to produce Mudanpi, Baishao, and Chishao. However, the roots are often harvested in autumn during the actual production in consideration of the dry matter accumulation. In addition, the level of paeoniflorin in the roots also varies throughout the day, increasing with temperature (Chen et al., 2002). This founding shows that it is better to harvest the roots at noon. Unlike the change of paeoniflorin in the roots, paeoniflorin in the leaves regularly decreased with plant development (Jian et al., 2010; Zhang et al., 2019b; Fu et al., 2020). The roots of Paeoniaceae plants usually can be used as medicines after four to five years of cultivation. However, the leaves can be harvested every year, so they can be developed as a new resource of paeoniflorin. Besides, removing some leaves at an early stage can reduce the incidence of plant disease, which is also much more conducive to the healthy growth of Paeoniaceae plants.

#### Organs

Paeoniflorin has been widely found in various organs of Paeoniaceae plants, but its contents were significantly different (Supplementary Table 4). The paeoniflorin content in *P. lactiflora*, from high to low, was in the following order: rhizomes, roots, leaves, stems, and flowers (Hu et al., 2000). Yang et al. (2001) determined that the paeoniflorin content in *Paeonia rockii* was in the following order: branch bark, root phloem, fibrous root, leaf, and petiole. However, among the organs of *Paeonia delavayi* var. *lutea*, the highest paeoniflorin content is found in the fruit, followed by the leaf, cork, cortex, stem, and xylem (Feng et al., 2012). The paeoniflorin distribution in *P. ostii* differs from these results, with the highest paeoniflorin content in the leaves, followed by stems, petioles, petals, xylem, and phloem (Zhang et al., 2019b). During the production of Mudanpi, Baishao, and Chishao, only the roots are harvested, while the other organs of Paeoniaceae plants are discarded. These studies indicate that the other organs also have potential medicinal value due to the considerable amounts of paeoniflorin.

Paeoniflorin content was also different in different root tissues. The paeoniflorin content in the rhizome of *P. lactiflora* is close to or even higher than that in the root, and the content in the branch roots and fine roots is higher than that in the main roots and thick roots, respectively (Hu et al., 2000; Chen et al., 2002; Kang, 2011; Ji et al., 2014). The paeoniflorin content in the phloem (2.69%) of *P. lactiflora* is similar to that in the xylem (2.90%), but it is significantly lower than that in the cork periderm (4.65%; Wu and Zhao, 2005). Based on high mass resolution matrix-assisted laser desorption/ionization MS imaging, Li et al. (2021b) reported that paeoniflorin was observed mainly in the cork and phloem region of *P. suffruticosa* and in the xylem rays of *P. lactiflora*, showing a great contrast with wedge-shaped xylem patches. The distribution of paeoniflorin in the roots of most wild species belonging to Sect. *Moutan* is similar to that of *P. suffruticosa*. In contrast, paeoniflorin is mainly distributed in the xylem of *P. ostii*, regardless of whether the roots are from plants grown in wild or cultivated habitats (Zhang et al., 2019b). This finding also indicates that the root xylem should be fully utilized for medicinal purposes instead of being discarded.

#### Abiotic factors

In addition to biological factors, some abiotic factors, including the annual average temperature, annual precipitation, annual sunshine duration, annual total solar radiation, and soil type of the plant habitat, also affected the paeoniflorin content (Hu et al., 2000; Han et al., 2008; Yuan et al., 2020; Zhang, 2020). There is a significant positive correlation between the paeoniflorin content in the roots and annual precipitation (Zhou et al., 2007), but a significant negative correlation has been observed between the paeoniflorin content in the roots and the annual average temperature (Yuan et al., 2020). Moreover, the elements zinc and potassium in the soil also play positive roles in paeoniflorin synthesis (Chen et al., 2009). These results reveal that the ecological and environmental factors of habitat should be carefully considered in order to produce the traditional Chinese medicines with high paeoniflorin content. Boiling and drying are usually necessary steps in the production of Mudanpi, Baishao, and Chishao, and these measures are considered to have effects on paeoniflorin content. High temperatures and long-term heating account for the decrease in paeoniflorin content due to its poor thermostability (Jin et al., 2015; Huo et al., 2017; Shen et al., 2019; Zhang et al., 2019b), and thermostability is negatively correlated with the water content of the roots (Yan et al., 2003). These studies show that the freeze-drying is better suited for increasing paeoniflorin contents in the production of traditional Chinese medicines.

## Paeoniflorin biosynthesis in Paeoniaceae

### Characterization of the complete pathway

**Figure 1:**
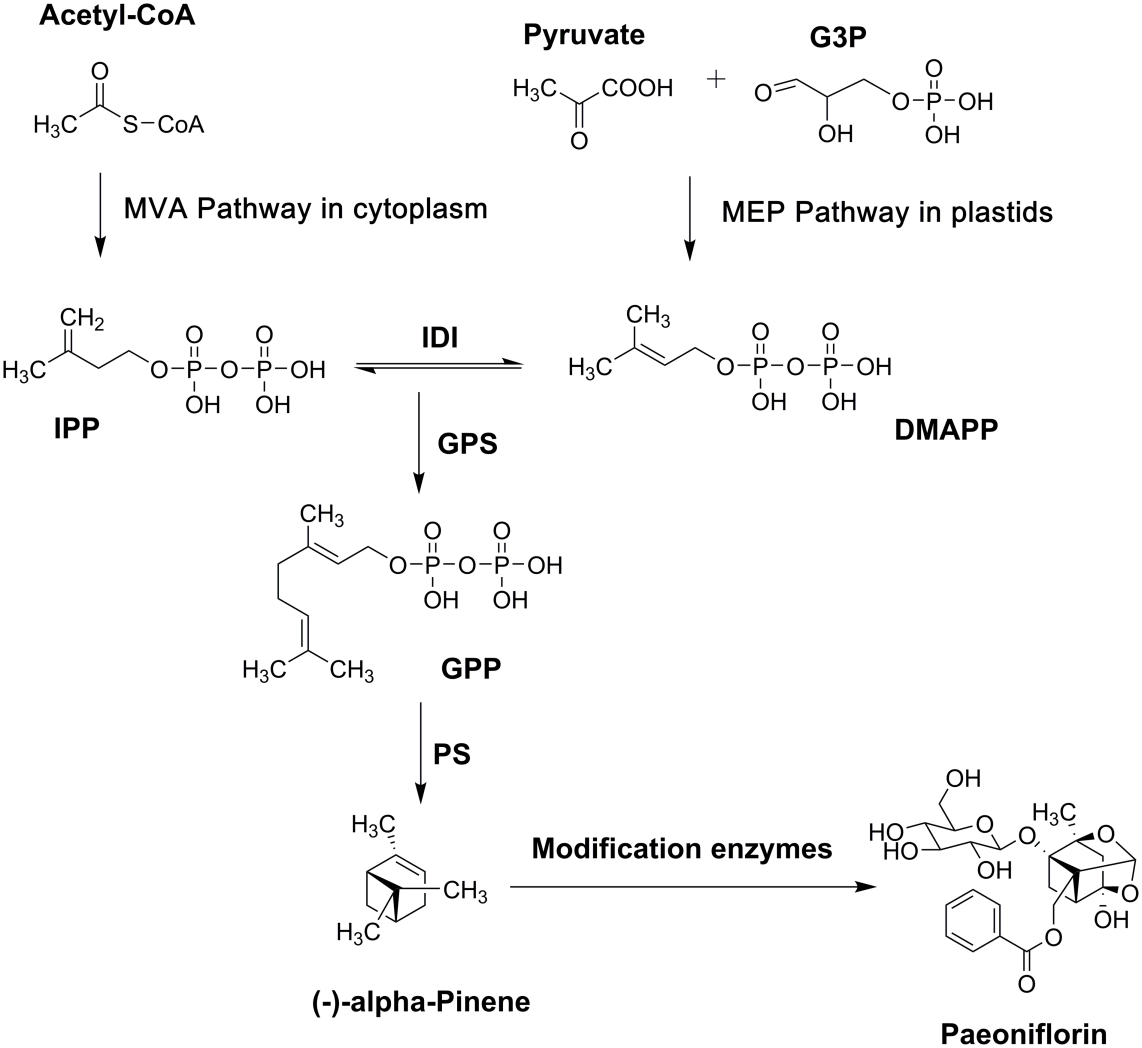
Biosynthesis pathway of paeoniflorin. G3P, glyceraldehyde-3-phosphate; MVA, mevalonate; MEP, 2-C-methyl-D-erythritol4-phosphate; IPP, isopentenyl pyrophosphate; DMAPP, dimethylallyl diphosphate; IDI, isopentenyl diphosphate isomerase; GPS, geranylgeranyl pyrophosphate synthase; GPP, geranyl diphosphate; PS, pinene synthase.

Paeoniflorin is a monoterpene glycoside, and its biosynthesis pathway can be divided into three stages (Figure 1). The universal precursor, isopentenyl pyrophosphate (IPP), and its isomer, dimethylallyl diphosphate (DMAPP), are synthesized in the first stage. Plants have two pathways for producing IPP and DMAPP. One is the mevalonate (MVA) pathway located in the cytoplasm with acetyl CoA as the raw material (Bekkering et al., 2018), and the other is the 2-C-methyl-D-erythritol4-phosphate (MEP) pathway, or 1-deoxy-D-xylulose5-phosphate (DXP) pathway, which is located in plastids and uses pyruvate and glyceraldehyde-3-phosphate as raw materials (Shi et al., 2019). These two pathways do not exist in isolation, and there is a universal interaction between them (Laule et al., 2003). The paeoniflorin content has been determined in different organs of *P. lactiflora* (Yuan et al., 2013), and the relative expression of 24 genes encoding enzymes involved in MEP/DXP and MVA pathways has been revealed. Correlation analysis indicates that hydroxymethylglutaryl-CoA synthase and phosphomevalonate kinase in the MVA pathway play important roles in the biosynthesis of paeoniflorin. In the second stage, geranyl diphosphate (GPP), the monoterpene precursor, is formed by head-to-tail condensation of one IPP and one DMAPP catalyzed by geranyl diphosphate synthase in the plastids (Zebec et al., 2016).

Then, the formation and post-modification of the basic pinene skeleton occur in the final stage. Terpenoid synthase (TPS), located at the branch point of the isoprenoid pathway, catalyzes GPP to form the monoterpenoid skeleton directly. Therefore, TPS is not only the key enzyme for terpenoid synthesis but also the main inducer for the structural diversity of terpenoids (Chen et al., 2011). A TPS gene named *PlPIN*, which encodes *α*-pinene synthase, was isolated from *P. lactiflora* (Ma et al., 2016). An *in vitro* enzyme assay showed that PlPIN converted GPP into *α*-pinene as a single product. Terpenoids in plants are often not the direct products obtained by the one-step catalysis of TPS, and most of them must be modified by special groups, even if their skeleton structures are rearranged. These post-modifications, including glycosylation, acylation, epoxidation, hydroxylation, halogenation, and addition and reduction reactions, substantially increase the variety of terpenoids and their structural diversity (Rivas et al., 2013; Zhao et al., 2014; Li et al., 2015). Unfortunately, no studies have addressed the post-modification of paeoniflorin biosynthesis.

### Hypothesized pathways of post-modification

**Figure 2:**
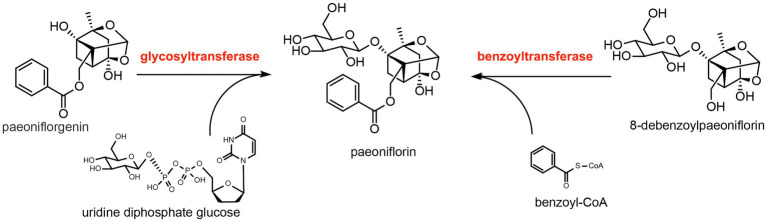
Predicted synthesis of paeoniflorin catalyzed by glycosyltransferase and benzoyltransferase. The substrate of glycosyltransferase is paeoniflorgenin and the glycosyl donor is uridine diphosphate glucose. The substrate of benzoyltransferase is 8-debenzoylpaeoniflorin and the benzoyl donor is benzoyl-CoA.

The basic skeleton of paeoniflorin is *α*-pinane, which is also connected to the benzoyl group, *β*-D-glucopyranosyl group, and hemiketal–acetal linkage. Based on previous literature (Chedgy et al., 2015; Xu et al., 2016), we speculated that the *β*-D-glucopyranosyl group is connected to the skeleton through glycosylation, which is catalyzed by glycosyltransferase, while the benzoyl group is connected to the skeleton through acylation, which is catalyzed by benzoyltransferase (Figure 2).

**Figure 3:**
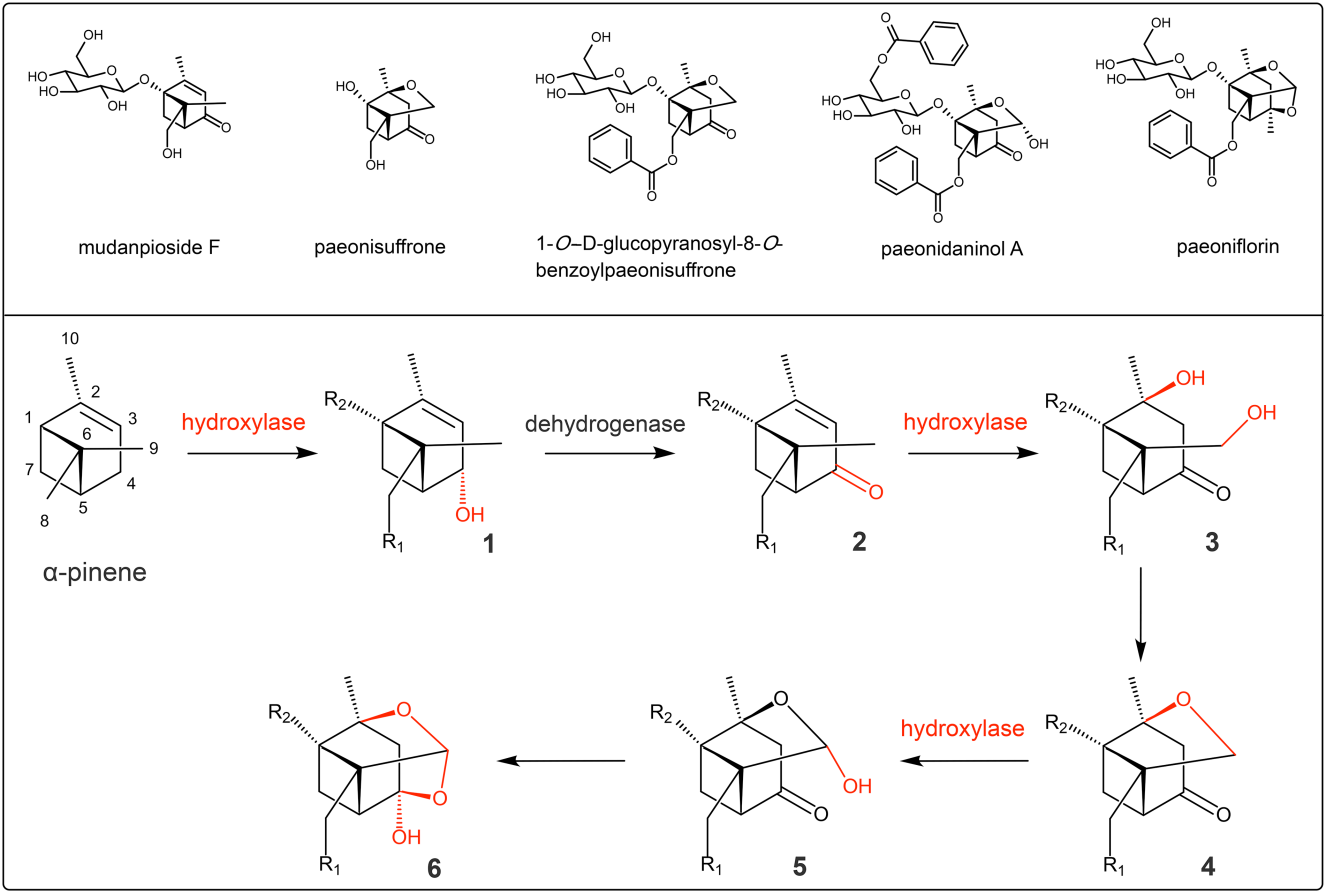
Predicted biosynthesis pathway of the hemiketal–acetal linkage of paeoniflorin. Compounds 1 and 3 are possible intermediates; the representative of compound 2 is mudanpioside F; the representatives of compound 4 are paeonisuffrone and 1-*O*-D-glucopyranosyl-8-*O*-benzoylpaeonisuffrone; the representative of compound 5 is paeonidaninol A; the representative of compound 6 is paeoniflorin; R_1_ and R_2_ represent the possible modification groups.

Unfortunately, there is a lack of sufficient understanding of the biosynthetic mechanism of the hemiketal–acetal linkage. Based on the compounds with structures similar to paeoniflorin in Paeoniaceae (He et al., 2010; Wu et al., 2010), the biosynthesis pathway of hemiketal-acetal linkage is proposed for the first time in the present study (Figure 3). First, the C-4 of *α*-pinene is hydroxylated by hydroxylase and then oxidized to the ketone group by dehydrogenase. Second, C-2 and C-9 are both hydroxylated by hydroxylases, and then, the two hydroxyl groups dehydrate to form a cyclic ether linkage. Finally, C-9 continues to be added to the hydroxyl group under the catalysis of hydroxylase, at which time the hemiacetal linkage is formed. Then, the hydroxyl group of C-9 reacts with the ketone group to form the hemiketal, while the hemiacetal is converted into acetal, eventually forming the hemiketal–acetal linkage. In these reaction steps, the hydroxylation of C-2, C-4, and C-9 is the basis.

Combining the reaction process in Figure 2 and the structure of *α*-pinene (Figure 3), it can be shown that the glycosylation modification is the reaction between the glycosyl donor (uridine diphosphate glucose) and the hydroxyl group at C-1, and the acylation modification is the reaction between the benzoyl donor (benzoyl-CoA) and the hydroxyl group at C-8. In these reaction steps, the hydroxylation of C-1 and C-8 is the basis. In summary, the hydroxylation of C1, C2, C4, C8, and C9 is the basic and necessary step in the modification stages of paeoniflorin biosynthesis, and hydroxylases play crucial roles in these stages.

## Discussion

High-throughput sequencing has been increasingly applied to the mining of key enzyme genes for natural product biosynthesis. By using plant tissues with large differences in natural product content as materials, many differentially expressed genes (DEGs) can be obtained by transcriptome sequencing, and the candidate enzyme genes can be further obtained based on a comparison with online databases, such as non-redundant protein sequences, Kyoto Encyclopedia of Genes and Genomes, and Gene Ontology. Plant materials are crucial throughout the sequencing process and directly determine the number of DEGs under the established analysis process. The plant materials for sequencing should preferably be based on the univariate principle, for example, the same tissues with different developmental stages, and whether the same tissues experience hormone induction or adversity stress. *PlPIN*, the *α*-pinene synthase gene, was isolated using the transcriptome data of *P. lactiflora* roots (Ma et al., 2016). Paeoniflorin content in the leaves of *P. ostii* is significantly correlated with developmental stage, with the paeoniflorin content being higher at earlier developmental stages. These differences in paeoniflorin content make the leaf a more suitable sequencing material for a detailed investigation of the biosynthesis pathway (Zhang et al., 2019b).

The functions of candidate genes obtained by transcriptome sequencing can be quickly identified by enzyme assays *in vitro*. However, for plant studies, it is usually insufficient to verify the functions of candidate genes through enzyme assays *in vitro*, and the functions of candidate genes in plants need to be further verified. Paeoniflorin is the characteristic constituent of Paeoniaceae; thus, it is almost impossible to verify the biological functions of candidate genes in model plants (e.g., *Arabidopsis* and *Petunia*). Additionally, at least at present, the long-life cycle of Paeoniaceae and the immature genetic transformation system make it difficult to overexpress candidate genes in herbaceous peony or tree peony to verify their biological functions. Fortunately, the virus-induced gene silencing system has been established in tree peony (Xie et al., 2019; Wang et al., 2020), which can be used to verify their biological functions by transiently silencing the candidate genes *in vivo*.

Analysis of the paeoniflorin biosynthesis pathway is still at the preliminary stage, and many scientific questions are yet to be answered. At present, the most urgent task is the identification and functional analysis of key modification enzyme genes. There are several studies on the generation of *β*-D-glucopyranosyl and benzoyl groups in natural products, which can provide a reference for paeoniflorin biosynthesis. It is believed that the identification and functional analysis of glycosyltransferase and benzoyltransferase genes will be completed in a short time. Nevertheless, the biosynthesis of the hemiketal–acetal linkage has not yet been reported, and we speculate that there are several enzymes involved in this pathway, including multi-step reactions, which may be the key restricting the analysis of paeoniflorin biosynthesis. With the development of technology, some new techniques, such as a target identification strategy based on biosynthetic intermediate probes, have been successfully used to discover key enzymes (Gao et al., 2020). We speculate that a breakthrough will be achieved in the elucidation of paeoniflorin biosynthesis in the near future. This will not only provide technical support to produce paeoniflorin using synthetic biology but will also lay the theoretical foundation for the use of biotechnology to improve the quality of Paeoniaceae.

## Author contributions

X-XZ, Y-HH, and J-HY conceived and designed this review. X-XZ wrote the manuscript. J-QZ, Y-TW, and H-YD performed the literature collection and made classification. J-HY and Y-HH edited and revised the manuscript. All authors contributed to the article and approved the submitted version.

## Funding

This work was supported by the National Natural Science Foundation of China (32000240), Key project at central government level, the ability establishment of sustainable use for valuable Chinese medicine resources (2060302), Chinese Universities Scientific Fund (2452020213), Open Project of Shanghai Key Laboratory of Plant Functional Genomics and Resources (PFGR202203), and Special Fund for Shanghai Landscaping Administration Bureau Program (G222415, G192418, G192419).

## Conflict of interest

The authors declare that the research was conducted in the absence of any commercial or financial relationships that could be construed as a potential conflict of interest.

## Publisher’s note

All claims expressed in this article are solely those of the authors and do not necessarily represent those of their affiliated organizations, or those of the publisher, the editors and the reviewers. Any product that may be evaluated in this article, or claim that may be made by its manufacturer, is not guaranteed or endorsed by the publisher.
