# Supplementary material for: Paeoniflorin in Paeoniaceae: Distribution, influencing factors, and biosynthesis
Source: Front Plant Sci. 2022 Sep 2;13:980854. doi: 10.3389/fpls.2022.980854 (PMC9478390; doi:10.3389/fpls.2022.980854)
Supplement: Supplementary file 1 [file Table_1.docx]

Supplementary Material

**Supplementary Table 1. Paeoniflorin content in the roots of different species belonging to Paeoniaceae.**

| **Species** | **Collection site** | **Collection date (Developmental stage)** | **Method** | **Content (mg/g)** | **References** |
| --- | --- | --- | --- | --- | --- |
| *Paeonia suffruticosa* | Hefei, Anhui | March 20 (Nutritional period) | TLC | 12.6* | He et al., 1980 |
| *P. suffruticosa* | Jinan, Shandong | March 20 (Nutritional period) | TLC | 9.0* | He et al., 1980 |
| *P. suffruticosa* | Beijing | \ | TLC | 20.2* | Yu and Xiao, 1987 |
| *P. suffruticosa* | Shaanxi | \ | TLC | 13.7* | Yu and Xiao, 1987 |
| *P. suffruticosa* | Xi'an, Shaanxi | \ | TLC | 25.0* | Yu and Xiao, 1987 |
| *P. suffruticosa* | Sichuan | \ | TLC | 17.7* | Yu and Xiao, 1987 |
| *P. suffruticosa* | Hunan | \ | TLC | 9.1* | Yu and Xiao, 1987 |
| *P. suffruticosa* | Geneva | \ | TLC | 19.9* | Yu and Xiao, 1987 |
| *P. suffruticosa* | Beijing (Introduced from Heze, Shandong) | October 25 | HPLC | 25.6* | Guo et al., 2002 |
| *P. suffruticosa* | Beijing | October 25 | HPLC | 14.1* | Guo et al., 2002 |
| *Paeonia ostii* | Beijing (Introduced from Tongling, Anhui) | October 25 | HPLC | 17.4* | Guo et al., 2002 |
| *P. ostii* | Lushi, Henan | September to October | UPLC-MS | 7.6 | Zhang et al., 2019b |
| *P. ostii* | Lushi, Henan | August to September | HPLC-MS | 6.5 | Yan et al., 2021 |
| *P. ostii* | Feng, Shaanxi | August to September | HPLC-MS | 5.8 | Yan et al., 2021 |
| *Paeonia qiui* | Xunyang, Shaanxi | September to October | UPLC-MS | 14.0 | Zhang et al., 2019b |
| *P. qiui* | Xunyang, Shaanxi | August to September | HPLC-MS | 24.2 | Yan et al., 2021 |
| *Paeonia rockii* | Beijing | October 25 | HPLC | 13.7* | Guo et al., 2002 |
| *P. rockii* | Beijing (Introduced from Taibai Mountain) | October 25 | HPLC | 46.8* | Guo et al., 2002 |
| *P. rockii* | Feng County, Shaanxi | September to October | UPLC-MS | 8.7 | Zhang et al., 2019b |
| *P.rockii* ssp. *rockii* | Taibai, Gansu | August to September | HPLC-MS | 6.0 | Yan et al., 2021 |
| *Paeonia jishanensis* | Yichuan, Shaanxi | September to October | UPLC-MS | 19.6 | Zhang et al., 2019b |
| *P. jishanensis* | Yan’an, Shaanxi | August to September | HPLC-MS | 17.2 | Yan et al., 2021 |
| *Paeonia decomposita* | Jinchuan, Sichuan | \ | HPLC | 48.2* | Guo et al., 2002 |
| *P. decomposita* | Heishui, Sichuan | \ | HPLC | 43.4* | Guo et al., 2002 |
| *P. decomposita* | Maerkang, Sichuan | September to October | UPLC-MS | 12.3 | Zhang et al., 2019b |
| *P. decomposita* | Maerkang, Sichuan | August to September | HPLC-MS | 14 | Yan et al., 2021 |
| *Paeonia rotundiloba* | Li County, Sichuang | August to September | HPLC-MS | 9.8 | Yan et al., 2021 |
| *Paeonia szechuanica* | Sichuan | \ | TLC | 7.9* | Yu and Xiao, 1987 |
| *P. szechuanica* | Jinchuan, Sichuan | \ | TLC | 18.8* | Yu and Xiao, 1987 |
| *Paeonia delavayi* | Lijiang, Yunnan | July 17 (Fruiting) | TLC | 19.3* | He et al., 1980 |
| *P. delavayi* | Yunnan | \ | TLC | 7.8* | Yu and Xiao, 1987 |
| *P. delavayi* | Sichuan | \ | TLC | 10.9* | Yu and Xiao, 1987 |
| *P. delavayi* | Yajiang, Sichuan | September to October | UPLC-MS | 6.2 | Zhang et al., 2019b |
| *P. delavayi* | Chengjiang, Yunnan | September to October | UPLC-MS | 1.9 | Zhang et al., 2019b |
| *P. delavayi* | Lanping, Yunnan | September to October | UPLC-MS | 4.5 | Zhang et al., 2019b |
| *P. delavayi* | Yulong, Yunnan | September to October | UPLC-MS | 6.1 | Zhang et al., 2019b |
| *P. delavayi* | Lanping, Yunnan | August to September | HPLC-MS | 16.2 | Yan et al., 2021 |
| *P. delavayi* var. *lutea* | Dali, Yunnan, | July 17 (Fruiting) | TLC | 16.1* | He et al., 1980 |
| *P. delavayi* var. *lutea* | Kunming, Yunnan, | May 15 (flowering) | TLC | 14.5* | He et al., 1980 |
| *P. delavayi* var. *lutea* | Tibet | July 17 (Fruiting) | TLC | 25.2* | He et al., 1980 |
| *P. delavayi* var. *lutea* | Nanchang, Sichuan | March 20 (Nutritional period) | TLC | 21.7* | He et al., 1980 |
| *P. delavayi* var. *lutea* | Chengdu, Sichuan | \ | TLC | 14.2* | Yu and Xiao, 1987 |
| *P. delavayi* var. *lutea* | Aba, Sichuan | \ | TLC | 18.5* | Yu and Xiao, 1987 |
| *P. delavayi* var. *lutea* | Tibet | \ | TLC | 17.2* | Yu and Xiao, 1987 |
| *P. delavayi* var. *lutea* | Dali, Yunnan | March 22 | RT-HPLC | 7.6* | Jin et al., 1989 |
| *P. delavayi* var. *lutea* | Dali, Yunnan | August 18 | RT-HPLC | 18.2* | Jin et al., 1989 |
| *Paeonia ludlowii* | Linzhi, Tibet | September to October | UPLC-MS | 2.3 | Zhang et al., 2019b |
| *P. ludlowii* | Linzhi, Tibet | August to September | HPLC-MS | 0.9 | Yan et al., 2021 |
| *Paeonia lactiflora* | Fengcheng, Liaoning | March 20 (Nutritional period) | TLC | 70.2* | He et al., 1980 |
| *P. lactiflora* | Inner Mongolia | March 20 (Nutritional period) | TLC | 64.0* | He et al., 1980 |
| *P. lactiflora* | Hangzhou, Zhejiang | March 20 (Nutritional period) | TLC | 50.8* | He et al., 1980 |
| *P. lactiflora* | Xuanhua, Hebei | March 20 (Nutritional period) | TLC | 49.6* | He et al., 1980 |
| *P. lactiflora* | Jiutai, Jilin | May 1 (Budding) | TLC | 43.4* | He et al., 1980 |
| *P. lactiflora* | Linjiang, Jilin | May 15 (flowering) | TLC | 47.0* | He et al., 1980 |
| *P. lactiflora* | Huairou, Hebei | July 17 (Fruiting) | TLC | 34.0* | He et al., 1980 |
| *P. lactiflora* | Xinglong, Hebei | May 1 (Budding) | TLC | 107.2* | He et al., 1980 |
| *P. lactiflora* | Beijing | \ | TLC | 28.8* | Yu and Xiao, 1987 |
| *P. lactiflora* | Liaoning | \ | TLC | 36.9* | Yu and Xiao, 1987 |
| *P. lactiflora* | Heilongjiang | \ | TLC | 43.6* | Yu and Xiao, 1987 |
| *P. lactiflora* | Geneva | \ | TLC | 28.1* | Yu and Xiao, 1987 |
| *P. lactiflora* | Mishan, Heilongjiang | July 16 | RT-HPLC | 75.5* | Jin et al., 1989 |
| *P. lactiflora* | Ning’an, Heilongjiang | July 16 | RT-HPLC | 62.8* | Jin et al., 1989 |
| *P. lactiflora* | Qiangguo, Jilin | June 15 | RT-HPLC | 54.5* | Jin et al., 1989 |
| *P. lactiflora* | Emei, Sichuan | July 2 | RT-HPLC | 23.3* | Jin et al., 1989 |
| *P. lactiflora* | Lveyang, Shaanx | September to October | UPLC-MS | 29.2 | Zhang et al., 2019b |
| *P. lactiflora* | Kangding, Sichuan | August 16 | HPLC-DAD, HPLC-Q-TOF-MS | 22.2 | Yang et al., 2020 |
| *P. lactiflora* | Genhe, Inner Mongolia | September 15 | HPLC-DAD, HPLC-Q-TOF-MS | 30.8 | Yang et al., 2020 |
| *P. lactiflora* | Hulun Buri Meng, Inner Mongolia | September 15 | HPLC-DAD, HPLC-Q-TOF-MS | 24.3 | Yang et al., 2020 |
| *P. lactiflora* | Luanchuan, Henan | August 25 | HPLC-DAD, HPLC-Q-TOF-MS | 39.29 | Yang et al., 2020 |
| *P. lactiflora* | Ning Co., Inner Mongolia | August to September | HPLC-MS | 77.2 | Yan et al., 2021 |
| *P. lactiflora* | Lveyang, Shaanxi | August to September | HPLC-MS | 70.7 | Yan et al., 2021 |
| *P. lactiflora* | Keshiketeng, Inner Mongolia | August to September | HPLC-MS | 81.0 | Yan et al., 2021 |
| *P. lactiflora* | Balinzuo, Inner Mongolia | August to September | HPLC-MS | 92.3 | Yan et al., 2021 |
| *P. lactiflora* | Qiqihar, Heilongjiang | August to September | HPLC-MS | 86.3 | Yan et al., 2021 |
| *P. lactiflora* var. *trichocarpa* | Hangzhou, Zhejiang | March 20(Nutritional period) | TLC | 57.0* | He et al., 1980 |
| *P. lactiflora* var*. trichocarpa* | Beijing | March 20(Nutritional period) | TLC | 49.6* | He et al., 1980 |
| *P. lactiflora* var. *trichocarpa* | Pan’an, Zhejiang | May 16 | RT-HPLC | 77.8* | Jin et al., 1989 |
| *P. lactiflora* var. *trichocarpa* | Bozhou, Anhui | May 15 | RT-HPLC | 64.5* | Jin et al., 1989 |
| *Paeonia veitchii* | Huzhu, Qinghai | March 20 (Nutritional period) | TLC | 57.6* | He et al., 1980 |
| *P. veitchii* | Minhe, Qinghai | July 17 (Fruiting) | TLC | 28.6* | He et al., 1980 |
| *P. veitchii* | Maowen, Sichuan | May 1 (Budding) | TLC | 43.2* | He et al., 1980 |
| *P. veitchii* | Yanchang, Gansu | May 15 (flowering) | TLC | 18.6* | He et al., 1980 |
| *P. veitchii* | Shanxi | \ | TLC | 13.7* | Yu and Xiao, 1987 |
| *P. veitchii* | Tibet | \ | TLC | 20.6* | Yu and Xiao, 1987 |
| *P. veitchii* | Qinghai | \ | TLC | 13.4* | Yu and Xiao, 1987 |
| *P. veitchii* | Maowen, Sichuan | June 15 | RT-HPLC | 41.3* | Jin et al., 1989 |
| *P. veitchii* | Songoan, Sichuan | May 14 | RT-HPLC | 52.1* | Jin et al., 1989 |
| *P. veitchii* | Minhe, Qinghai | August to September | HPLC-MS | 74.4 | Yuan et al., 2020 |
| *P. veitchii* | Lintao, Gansu | August to September | HPLC-MS | 71.3 | Yuan et al., 2020 |
| *P. veitchii* | Feng Co., Shanxi | August to September | HPLC-MS | 62.1 | Yuan et al., 2020 |
| *P. veitchii* | Daofu, Sichuan | August to September | HPLC-MS | 90.6 | Yuan et al., 2020 |
| *P. veitchii* | Longnan, Gansu | August to September | HPLC-MS | 30.7 | Yuan et al., 2020 |
| *P. veitchii* | MaerKang, Sichuan | August to September | HPLC-MS | 73.9 | Yuan et al., 2020 |
| *P. veitchii* | Weibin, Shaanxi | August to September | HPLC-MS | 39.1 | Yuan et al., 2020 |
| *P. veitchii* | Xiaojin, Sichuan | August 14 | HPLC-DAD, HPLC-Q-TOF-MS | 20.1 | Yang et al., 2020 |
| *P. veitchii* | Rangtang, Sichuan | August 15 | HPLC-DAD, HPLC-Q-TOF-MS | 12.1 | Yang et al., 2020 |
| *P. veitchii* | Yuzhong, Gansu | September 2 | HPLC-DAD, HPLC-Q-TOF-MS | 12.1 | Yang et al., 2020 |
| *P. veitchii* | Luhuo, Sichuan | August 16 | HPLC-DAD, HPLC-Q-TOF-MS | 26.1 | Yang et al., 2020 |
| *P. veitchii* var. *uniflora* | Tibet | \ | TLC | 34.7* | Yu and Xiao, 1987 |
| *P. veitchii* var. *woodwardii* | Geneva | \ | TLC | 9.6* | Yu and Xiao, 1987 |
| *P. veitchii* var. *woodwardii* | Wolong, Sichuan | July 12 | RT-HPLC | 33.8* | Jin et al., 1989 |
| *Paeonia obovata* | Baihua Mountain, Hebei | May 15 (flowering) | TLC | 21.6* | He et al., 1980 |
| *P. obovata* | Yangcheng, Shanxi | July 17 (Fruiting) | TLC | 10.6* | He et al., 1980 |
| *P. obovata* | Sichuan | March 20 (Nutritional period) | TLC | 24.6* | He et al., 1980 |
| *P. obovata* | Ji'an, Jilin | March 20 (Nutritional period) | TLC | 2.7* | He et al., 1980 |
| *P. obovata* | Beijing | \ | TLC | 13.7* | Yu and Xiao, 1987 |
| *P. obovata* | Sichuan | \ | TLC | 8.2* | Yu and Xiao, 1987 |
| *P. obovata* | Jinlin | \ | TLC | 6.7* | Yu and Xiao, 1987 |
| *P. obovata* | Zuojia, Jilin | May 26 | RT-HPLC | 14.7* | Jin et al., 1989 |
| *P. obovata* | Panshi, Jilin | September 20 | HPLC-DAD, HPLC-Q-TOF-MS | 0.5 | Yang et al., 2020 |
| *P. obovata* | Beijing | September 10 | HPLC-DAD, HPLC-Q-TOF-MS | 33.5 | Yang et al., 2020 |
| *P. obovata* ssp. *obovata* | Jiaohe, Jilin | September to October | UPLC-MS | 26.2 | Zhang et al., 2019b |
| *P. obovata* ssp. *obovata* | Jiaohe, Jilin | August to September | HPLC-MS | 7.4 | Yan et al., 2021 |
| *P. obovata* ssp. *obovata* | Shangzhi, Heilongjiang | August to September | HPLC-MS | 33.3 | Yan et al., 2021 |
| *P. obovata* var. *willmottiae* | Kang Co., Gansu | July 17(Fruiting) | TLC | 25.4* | He et al., 1980 |
| *P. obovata* var. *willmottiae* | Ruoerge, Sichuan | July 17(Fruiting) | TLC | 0.9* | He et al., 1980 |
| *P. obovata* var. *willmottiae* | Maowen, Sichuan | July 1 | RT-HPLC | 38.2* | Jin et al., 1989 |
| *P. obovata* ssp. *willmottiae* | Lveyang, Shaanx | September to October | UPLC-MS | 7.7 | Zhang et al., 2019b |
| *P. obovata* ssp*. willmottiae* | Lveyang, Shaanxi | August to September | HPLC-MS | 21.6 | Yan et al., 2021 |
| *P. obovata* ssp. *willottiae* | Kangding, Sichuan | August 16 | HPLC-DAD, HPLC-Q-TOF-MS | 9.6 | Yang et al., 2020 |
| *P. obovata* ssp. *willottiae* | Luanchuan, Henan | August 25 | HPLC-DAD, HPLC-Q-TOF-MS | 15.9 | Yang et al., 2020 |
| *P. obovata* ssp. *willottiae* | Wufeng, Hubei | September 18 | HPLC-DAD, HPLC-Q-TOF-MS | 6.9 | Yang et al., 2020 |
| *P. obovata* ssp. *willottiae* | Baokang, Hubei | September 5 | HPLC-DAD, HPLC-Q-TOF-MS | 12.2 | Yang et al., 2020 |
| *Paeonia emodi* | Geneva | \ | TLC | 5.6* | Yu and Xiao, 1987 |
| *P. emodi* | Jilong, Tibet | September to October | UPLC-MS | 37.8 | Zhang et al., 2019b |
| *P. emodi* | Jilong, Tibet | August 30 | HPLC-DAD, HPLC-Q-TOF-MS | 24.5 | Yang et al., 2020 |
| *P. emodi* | Jilong, Tibet | August to September | HPLC-MS | 51.7 | Yan et al., 2021 |
| *Paeonia sterniana* | Bomi, Tibet | September to October | UPLC-MS | 51.2 | Zhang et al., 2019b |
| *P. sterniana* | Bomi, Tibet | August 5 | HPLC-DAD, HPLC-Q-TOF-MS | 24.7 | Yang et al., 2020 |
| *P. stemiana* | Bomi, Tibet | August to September | HPLC-MS | 53.3 | Yan et al., 2021 |
| *Paeonia intermedia* | Yumin, Xinjiang | September to October | UPLC-MS | 3.1 | Zhang et al., 2019b |
| *P. intermedia* | Yumin, Xinjiang | August 15 | HPLC-DAD, HPLC-Q-TOF-MS | 11.7 | Yang et al., 2020 |
| *P. intermedia* | Yining, Xinjiang | August to September | HPLC-MS | 10.2 | Yan et al., 2021 |
| *P. intermedia* | Yumin, Xinjiang | August to September | HPLC-MS | 18.9 | Yan et al., 2021 |
| *Paeonia mairei* | Hongya, Sichuan | May 15 (flowering) | TLC | 27.2* | He et al., 1980 |
| *P. mairei* | Sichuan | \ | TLC | 26.6* | Yu and Xiao, 1987 |
| *P. mairei* | Maowen, Sichuan | June 9 | RT-HPLC | 14.0* | Jin et al., 1989 |
| *P. mairei* | Nanping, Sichuan | May 9 | RT-HPLC | 30.7* | Jin et al., 1989 |
| *P. mairei* | Xunyang, Shaanxi | September to October | UPLC-MS | 29.5 | Zhang et al., 2019b |
| *P. mairei* | Xunyang, Shaanxi | August to September | HPLC-MS | 22.8 | Yan et al., 2021 |
| *P. mairei* | Baoxing, Sichuan | August 18 | HPLC-DAD, HPLC-Q-TOF-MS | 14.5 | Yang et al., 2020 |
| *P. mairei* | Foping, Shanxi | September 8 | HPLC-DAD, HPLC-Q-TOF-MS | 26.7 | Yang et al., 2020 |
| *Paeonia anomala* | Geneva | \ | TLC | 13.0* | Yu and Xiao, 1987 |
| *P. anomala* | Tacheng, Xinjiang | June 3 | RT-HPLC | 26.2* | Jin et al., 1989 |
| *P. anomala* | Altay, Xinjiang | August 20 | HPLC-DAD, HPLC-Q-TOF-MS | 15.6 | Yang et al., 2020 |
| *P. anomala* ssp*. veitchii* | Feng Co, Shaanxi | September to October | UPLC-MS | 21.8 | Zhang et al., 2019b |
| *P. anomala* ssp. *veitchii* | Wudu, Gansu | September to October | UPLC-MS | 27.8 | Zhang et al., 2019b |
| *P. anomala* ssp. *veitchii* | Wudu, Gansu | August to September | HPLC-MS | 64.7 | Yan et al., 2021 |
| *P. anomala* ssp. *anomala* | Jimunai, Xinjiang | September to October | UPLC-MS | 6.9 | Zhang et al., 2019b |
| *P. anomala* ssp. *anomala* | Jimunai, Xinjiang | August to September | HPLC-MS | 57.0 | Yan et al., 2021 |
| *P. anomala* ssp. *anomala* | Kanas, Xinjiang | August to September | HPLC-MS | 51.1 | Yan et al., 2021 |
| *P. anomala* var. *intermedia* | Tianshan, Xinjiang | July 17 (Fruiting) | TLC | 14.0* | He et al., 1980 |
| *P. anomala* var. *intermedia* | Altai, Xinjiang | March 20 (Nutritional period) | TLC | 21.6* | He et al., 1980 |
| *P. anomala* var. *intermedia* | Xinyuan, Xinjiang | May 15 (flowering) | TLC | 24* | He et al., 1980 |
| *P. anomala* var. *intermedia* | Xinjiang | \ | TLC | 13.9* | Yu and Xiao, 1987 |
| *P. anomala* var. *intermedia* | Tacheng Co, Xinjiang | June 3 | RT-HPLC | 22.1* | Jin et al., 1989 |
| *Paeonia sinjiangensis* | Altai Co, Xinjiang | May 15 (flowering) | TLC | 14.8* | He et al., 1980 |
| *P. sinjiangensis* | Xinjiang Prov. | \ | TLC | 13.3* | Yu and Xiao, 1987 |
| *P. sinjiangensis* | Tacheng, Xinjiang | June 3 | RT-HPLC | 29.5* | Jin et al., 1989 |
| *Paeonia japonica* | Heilongjiang | \ | RT-HPLC | 11.1* | Jin et al., 1989 |
| *Paeonia officinalis* | Vienna | \ | TLC | 25.7* | Yu and Xiao, 1987 |
| *P. officinalis* | Geneva | \ | TLC | 16.1* | Yu and Xiao, 1987 |
| *Paeonia decora* | Geneva | \ | TLC | 21.6* | Yu and Xiao, 1987 |
| *P. decora* | Geneva | \ | TLC | 17.8* | Yu and Xiao, 1987 |
| *Paeonia tenuifolia* | Geneva | \ | TLC | 42.5* | Yu and Xiao, 1987 |
| *P. tenuifolia* | Geneva | \ | TLC | 23.1* | Yu and Xiao, 1987 |
| *Paeonia bakeri* | Geneva | \ | TLC | 16.2* | Yu and Xiao, 1987 |
| *Paeonia paradoxa* | Geneva | \ | TLC | 6.8* | Yu and Xiao, 1987 |
| *Paeonia humilis* var. *villose* | Geneva | \ | TLC | 14.4* | Yu and Xiao, 1987 |
| *Paeonia banatica* | Geneva | \ | TLC | 14* | Yu and Xiao, 1987 |
| *Paeonia corallina* | Geneva | \ | TLC | 8.3* | Yu and Xiao, 1987 |
| *P. coriacea* var. *atlantica* | Geneva | \ | TLC | 4* | Yu and Xiao, 1987 |

*The content in the original study was expressed as the percentage.

**Supplementary Table 2. Paeoniflorin content in Paeoniaceae plants of different ages.**

| **Species** | **Organ** | **Plant ages (years)** | **Method** | **Content (mg/g)** | **References** |
| --- | --- | --- | --- | --- | --- |
| *Paeonia ostii* | cortex | 1 | HPLC | 53.0* | Yan et al., 2019 |
| *P. ostii* | cortex | 2 | HPLC | 41.2* | Yan et al., 2019 |
| *P. ostii* | cortex | 3 | HPLC | 29.8* | Yan et al., 2019 |
| *P. ostii* | cortex | 4 | HPLC | 25.5* | Yan et al., 2019 |
| *P. ostii* | cortex | 5 | HPLC | 25.8* | Yan et al., 2019 |
| *P. ostii* | phloem | 1 | UPLC-MS | 12.5 | Zhang et al., 2019b |
| *P. ostii* | phloem | 2 | UPLC-MS | 18.7 | Zhang et al., 2019b |
| *P. ostii* | phloem | 3 | UPLC-MS | 14.4 | Zhang et al., 2019b |
| *P. ostii* | phloem | 4 | UPLC-MS | 21.1 | Zhang et al., 2019b |
| *P. ostii* | phloem | 5 | UPLC-MS | 14.8 | Zhang et al., 2019b |
| *P. ostii* | phloem | 6 | UPLC-MS | 13.7 | Zhang et al., 2019b |
| *P. ostii* | phloem | 7 | UPLC-MS | 17.9 | Zhang et al., 2019b |
| *P. ostii* | xylem | 1 | UPLC-MS | 14.7 | Zhang et al., 2019b |
| *P. ostii* | xylem | 2 | UPLC-MS | 21.3 | Zhang et al., 2019b |
| *P. ostii* | xylem | 3 | UPLC-MS | 16.3 | Zhang et al., 2019b |
| *P. ostii* | xylem | 4 | UPLC-MS | 24.9 | Zhang et al., 2019b |
| *P. ostii* | xylem | 5 | UPLC-MS | 22.0 | Zhang et al., 2019b |
| *P. ostii* | xylem | 6 | UPLC-MS | 19.4 | Zhang et al., 2019b |
| *P. ostii* | xylem | 7 | UPLC-MS | 23.1 | Zhang et al., 2019b |
| *Paeonia lactiflora* | root | 1 | TLC-UV | 20.3 | Huang et al., 2000 |
| *P. lactiflora* | root | 3 | TLC-UV | 31.3 | Huang et al., 2000 |
| *P. lactiflora* | root | 5 | TLC-UV | 37.6 | Huang et al., 2000 |
| *P. lactiflora* | root | 7 | TLC-UV | 41.5 | Huang et al., 2000 |
| *P. lactiflora* | root | 1 | TLC-UV | 10.4* | Zhang, 2000 |
| *P. lactiflora* | root | 2 | TLC-UV | 22.1* | Zhang, 2000 |
| *P. lactiflora* | root | More than 3 | TLC-UV | 35.7* | Zhang, 2000 |
| *P. lactiflora* | root | 1 | HPLC | 18.9* | Li et al., 2008 |
| *P. lactiflora* | root | 2 | HPLC | 21.8* | Li et al., 2008 |
| *P. lactiflora* | root | 3 | HPLC | 19.5* | Li et al., 2008 |
| *P. lactiflora* | root | 4 | HPLC | 19.0* | Li et al., 2008 |
| *P. lactiflora* | root | 5 | HPLC | 18.8* | Li et al., 2008 |
| *P. lactiflora* | root (seedlings) | 1 | RP-HPLC | 28.3* | Jian et al., 2010 |
| *P. lactiflora* | root (seedlings) | 4 | RP-HPLC | 68.6* | Jian et al., 2010 |
| *P. lactiflora* | root (seedlings) | 6 | RP-HPLC | 69.1* | Jian et al., 2010 |
| *P. lactiflora* | root (transplanted rhizome) | 1 | RP-HPLC | 11.9* | Jian et al., 2010 |
| *P. lactiflora* | root (transplanted rhizome) | 2 | RP-HPLC | 38.8* | Jian et al., 2010 |
| *P. lactiflora* | root (transplanted rhizome) | 3 | RP-HPLC | 34.3* | Jian et al., 2010 |
| *P. lactiflora* | root (transplanted rhizome) | 7 | RP-HPLC | 49.0* | Jian et al., 2010 |
| *P. lactiflora* | root | 2 | HPLC | 39.5* | Kang, 2011 |
| *P. lactiflora* | root | 3 | HPLC | 41.5* | Kang, 2011 |
| *P. lactiflora* | root | 4 | HPLC | 46.1* | Kang, 2011 |
| *P. lactiflora* | root | 5 | HPLC | 53.2* | Kang, 2011 |
| *P. lactiflora* | root | More than 5 | HPLC | 53.2* | Kang, 2011 |
| *P. lactiflora* | phloem | 1 | UPLC-MS | 37.7 | Zhang et al., 2019b |
| *P. lactiflora* | phloem | 2 | UPLC-MS | 44.6 | Zhang et al., 2019b |
| *P. lactiflora* | phloem | 3 | UPLC-MS | 34.7 | Zhang et al., 2019b |
| *P. lactiflora* | xylem | 1 | UPLC-MS | 53.9 | Zhang et al., 2019b |
| *P. lactiflora* | xylem | 2 | UPLC-MS | 59.1 | Zhang et al., 2019b |
| *P. lactiflora* | xylem | 3 | UPLC-MS | 48.4 | Zhang et al., 2019b |

*The content in the original study was expressed as the percentage.

**Supplementary Table 3. Paeoniflorin content in Paeoniaceae plants at different developmental stages.**

| **Species** | **Organ** | **Plant ages (years)** | **Collection date (Developmental stage)** | **Method** | **Content (mg/g)** | **References** |
| --- | --- | --- | --- | --- | --- | --- |
| *Paeonia suffruticosa* | root | \ | March 30 | TLC | 69.1* | Yu and Xiao, 1985 |
| *P. suffruticosa* | root | \ | April 15 | TLC | 29.9* | Yu and Xiao, 1985 |
| *P. suffruticosa* | root | \ | May 4 | TLC | 50.7* | Yu and Xiao, 1985 |
| *P. suffruticosa* | root | \ | May 18 | TLC | 65.3* | Yu and Xiao, 1985 |
| *P. suffruticosa* | root | \ | June 17 | TLC | 69.0* | Yu and Xiao, 1985 |
| *P. suffruticosa* | root | \ | July18 | TLC | 45.9* | Yu and Xiao, 1985 |
| *P. suffruticosa* | root | \ | August 18 | TLC | 25.6* | Yu and Xiao, 1985 |
| *P. suffruticosa* | root | \ | September 19 | TLC | 32.0* | Yu and Xiao, 1985 |
| *P. suffruticosa* | root | \ | October 20 | TLC | 49.3* | Yu and Xiao, 1985 |
| *P. suffruticosa* | root | \ | November 18 | TLC | 36.5* | Yu and Xiao, 1985 |
| *Paeonia ostii* | leaf | \ | January 22 | UPLC-MS | 151.8 | Zhang et al., 2019b |
| *P. ostii* | leaf | \ | February 10 | UPLC-MS | 144.4 | Zhang et al., 2019b |
| *P. ostii* | leaf | \ | March 8 | UPLC-MS | 119.4 | Zhang et al., 2019b |
| *P. ostii* | leaf | \ | March 23 | UPLC-MS | 54.8 | Zhang et al., 2019b |
| *P. ostii* | leaf | \ | April 5 | UPLC-MS | 24.0 | Zhang et al., 2019b |
| *P. ostii* | leaf | \ | May 5 | UPLC-MS | 10.7 | Zhang et al., 2019b |
| *P. ostii* | leaf | \ | May 31 | UPLC-MS | 4.1 | Zhang et al., 2019b |
| *P. ostii* | leaf | \ | July 20 | UPLC-MS | 4.0 | Zhang et al., 2019b |
| *P. ostii* | leaf | \ | September 21 | UPLC-MS | 3.6 | Zhang et al., 2019b |
| *Paeonia lactiflora* | root | 5 | March 20 | TLC-UV | 48.8* | He et al., 1980 |
| *P. lactiflora* | root | 5 | May 1 | TLC-UV | 85.8* | He et al., 1980 |
| *P. lactiflora* | root | 5 | May 15 | TLC-UV | 78.4* | He et al., 1980 |
| *P. lactiflora* | root | 5 | July 15 | TLC-UV | 49.7* | He et al., 1980 |
| *P. lactiflora* | root | 5 | September 15 | TLC-UV | 38.2* | He et al., 1980 |
| *P. lactiflora* | root | 6 | March 20 | TLC-UV | 56.3* | He et al., 1980 |
| *P. lactiflora* | root | 6 | May 1 | TLC-UV | 94.0* | He et al., 1980 |
| *P. lactiflora* | root | 6 | May 15 | TLC-UV | 63.8* | He et al., 1980 |
| *P. lactiflora* | root | 6 | July 15 | TLC-UV | 27.7* | He et al., 1980 |
| *P. lactiflora* | root | 6 | September 15 | TLC-UV | 40.4* | He et al., 1980 |
| *P. lactiflora* | root | 6 | November 21 | TLC-UV | 40.4* | He et al., 1980 |
| *P. lactiflora* | root | \ | March 30 | TLC | 31.9* | Yu and Xiao, 1985 |
| *P. lactiflora* | root | \ | April 15 | TLC | 22.7* | Yu and Xiao, 1985 |
| *P. lactiflora* | root | \ | May 4 | TLC | 41.2* | Yu and Xiao, 1985 |
| *P. lactiflora* | root | \ | May 18 | TLC | 60.3* | Yu and Xiao, 1985 |
| *P. lactiflora* | root | \ | June 17 | TLC | 55.9* | Yu and Xiao, 1985 |
| *P. lactiflora* | root | \ | July18 | TLC | 44.2* | Yu and Xiao, 1985 |
| *P. lactiflora* | root | \ | August 18 | TLC | 40* | Yu and Xiao, 1985 |
| *P. lactiflora* | root | \ | September 19 | TLC | 45.7* | Yu and Xiao, 1985 |
| *P. lactiflora* | root | \ | October 20 | TLC | 53.7* | Yu and Xiao, 1985 |
| *P. lactiflora* | root | \ | November 18 | TLC | 50.2* | Yu and Xiao, 1985 |
| *P. lactiflora* | root | 3 | January 20 | TLC-UV | 30.1 | Huang et al., 2000 |
| *P. lactiflora* | root | 3 | May 10 | TLC-UV | 37.5 | Huang et al., 2000 |
| *P. lactiflora* | root | 3 | August 5 | TLC-UV | 31.5 | Huang et al., 2000 |
| *P. lactiflora* | root | 4 | January 19 | HPLC | 31.3* | Jin et al., 2010 |
| *P. lactiflora* | root | 4 | March 23 | HPLC | 40.4* | Jin et al., 2010 |
| *P. lactiflora* | root | 4 | April 18 | HPLC | 56.0* | Jin et al., 2010 |
| *P. lactiflora* | root | 4 | May 15 | HPLC | 55.5* | Jin et al., 2010 |
| *P. lactiflora* | root | 4 | June 1 | HPLC | 27.5* | Jin et al., 2010 |
| *P. lactiflora* | root | 4 | July 20 | HPLC | 18.7* | Jin et al., 2010 |
| *P. lactiflora* | root | 4 | August 19 | HPLC | 27.6* | Jin et al., 2010 |
| *P. lactiflora* | root | 4 | September 8 | HPLC | 20.3* | Jin et al., 2010 |
| *P. lactiflora* | root | 4 | September 27 | HPLC | 30.0* | Jin et al., 2010 |
| *P. lactiflora* | root | 4 | October 30 | HPLC | 35.9* | Jin et al., 2010 |
| *P. lactiflora* | root | 4 | December 5 | HPLC | 32.6* | Jin et al., 2010 |
| *P. lactiflora* | root | \ | March 20 | HPLC | 66.4* | Jian et al., 2010 |
| *P. lactiflora* | root | \ | May 15 | HPLC | 55.2* | Jian et al., 2010 |
| *P. lactiflora* | root | \ | June 14 | HPLC | 62.6* | Jian et al., 2010 |
| *P. lactiflora* | root | \ | August 20 | HPLC | 68.8* | Jian et al., 2010 |
| *P. lactiflora* | root | \ | May 15 | HPLC | 58.9* | Fu et al., 2020 |
| *P. lactiflora* | root | \ | May 30 | HPLC | 58.4* | Fu et al., 2020 |
| *P. lactiflora* | root | \ | June 15 | HPLC | 37.3* | Fu et al., 2020 |
| *P. lactiflora* | root | \ | July 15 | HPLC | 28.9* | Fu et al., 2020 |
| *P. lactiflora* | root | \ | July 30 | HPLC | 28.9* | Fu et al., 2020 |
| *P. lactiflora* | root | \ | August 15 | HPLC | 25.8* | Fu et al., 2020 |
| *P. lactiflora* | root | \ | August 30 | HPLC | 24.9* | Fu et al., 2020 |
| *P. lactiflora* | root | \ | September 15 | HPLC | 23.5* | Fu et al., 2020 |
| *P. lactiflora* | root | \ | September 30 | HPLC | 31.1* | Fu et al., 2020 |
| *P. lactiflora* | root | \ | October 15 | HPLC | 34.0* | Fu et al., 2020 |
| *P. lactiflora* | root | \ | October 30 | HPLC | 24.9* | Fu et al., 2020 |
| *P. lactiflora* var. *trichocarpa* | root | 4 | January 16 | RP-HPLC | 38.2* | Jin et al., 1991 |
| *P. lactiflora* var. *trichocarpa* | root | 4 | January 30 | RP-HPLC | 34.5* | Jin et al., 1991 |
| *P. lactiflora* var. *trichocarpa* | root | 4 | February 11 | RP-HPLC | 42.5* | Jin et al., 1991 |
| *P. lactiflora* var. *trichocarpa* | root | 4 | March 3 | RP-HPLC | 37.0* | Jin et al., 1991 |
| *P. lactiflora* var. *trichocarpa* | root | 4 | March 17 | RP-HPLC | 43.2* | Jin et al., 1991 |
| *P. lactiflora* var. *trichocarpa* | root | 4 | March 29 | RP-HPLC | 35.5* | Jin et al., 1991 |
| *P. lactiflora* var. *trichocarpa* | root | 4 | April 13 | RP-HPLC | 34.8* | Jin et al., 1991 |
| *P. lactiflora* var. *trichocarpa* | root | 4 | April 22 | RP-HPLC | 60.6* | Jin et al., 1991 |
| *P. lactiflora* var. *trichocarpa* | root | 4 | May 2 | RP-HPLC | 82.0* | Jin et al., 1991 |
| *P. lactiflora* var. *trichocarpa* | root | 4 | May 15 | RP-HPLC | 64.5* | Jin et al., 1991 |
| *P. lactiflora* var. *trichocarpa* | root | 4 | May 25 | RP-HPLC | 49.9* | Jin et al., 1991 |
| *P. lactiflora* var. *trichocarpa* | root | 4 | June 10 | RP-HPLC | 34.5* | Jin et al., 1991 |
| *P. lactiflora* var. *trichocarpa* | root | 4 | June 25 | RP-HPLC | 26.5* | Jin et al., 1991 |
| *P. lactiflora* var. *trichocarpa* | root | 4 | July 7 | RP-HPLC | 28.7* | Jin et al., 1991 |
| *P. lactiflora* var. *trichocarpa* | root | 4 | July 18 | RP-HPLC | 34.0* | Jin et al., 1991 |
| *P. lactiflora* var. *trichocarpa* | root | 4 | July 27 | RP-HPLC | 27.8* | Jin et al., 1991 |
| *P. lactiflora* var. *trichocarpa* | root | 4 | August 9 | RP-HPLC | 24.7* | Jin et al., 1991 |
| *P. lactiflora* var. *trichocarpa* | root | 4 | August 22 | RP-HPLC | 25.1* | Jin et al., 1991 |
| *P. lactiflora* var. *trichocarpa* | root | 4 | September 4 | RP-HPLC | 41.5* | Jin et al., 1991 |
| *P. lactiflora* var. *trichocarpa* | root | 4 | September 15 | RP-HPLC | 40.5* | Jin et al., 1991 |
| *P. lactiflora* var. *trichocarpa* | root | 4 | September 30 | RP-HPLC | 31.1* | Jin et al., 1991 |
| *P. lactiflora* var. *trichocarpa* | root | 4 | October 11 | RP-HPLC | 32.2* | Jin et al., 1991 |
| *P. lactiflora* var. *trichocarpa* | root | 4 | October 23 | RP-HPLC | 30.7* | Jin et al., 1991 |
| *P. lactiflora* var. *trichocarpa* | root | 4 | November 3 | RP-HPLC | 33.4* | Jin et al., 1991 |
| *P. lactiflora* var. *trichocarpa* | root | 4 | November 16 | RP-HPLC | 34.1* | Jin et al., 1991 |
| *P. lactiflora* var. *trichocarpa* | root | 4 | November 27 | RP-HPLC | 26.1* | Jin et al., 1991 |
| *P. lactiflora* var. *trichocarpa* | root | 4 | December 26 | RP-HPLC | 31.7* | Jin et al., 1991 |

*The content in the original study was expressed as the percentage.

**Supplementary Table 4.** Paeoniflorin content in the different organs of Paeoniaceae plants.

| **Species** | **Organ** | **Collection site** | **Collection date (Developmental stage)** | **Method** | **Content (mg/g)** | **References** |
| --- | --- | --- | --- | --- | --- | --- |
| *Paeonia ostii* | petal | Shanghai | February 10 | UPLC-MS | 37.5 | Zhang et al., 2019b |
| *P. ostii* | leaf | Shanghai | February 10 | UPLC-MS | 146.0 | Zhang et al., 2019b |
| *P. ostii* | petiole | Shanghai | February 10 | UPLC-MS | 40.9 | Zhang et al., 2019b |
| *P. ostii* | stem | Shanghai | February 10 | UPLC-MS | 54.4 | Zhang et al., 2019b |
| *P. ostii* | phloem | Shanghai | February 10 | UPLC-MS | 18.9 | Zhang et al., 2019b |
| *P. ostii* | xylem | Shanghai | February 10 | UPLC-MS | 20.2 | Zhang et al., 2019b |
| *P. ostii* | big root | Anhui | November 12 | HPLC | 6.3* | Ji et al., 2014 |
| *P. ostii* | medium root | Anhui | November 12 | HPLC | 8.1* | Ji et al., 2014 |
| *P. ostii* | small root | Anhui | November 12 | HPLC | 10.0* | Ji et al., 2014 |
| *Paeonia ludlowii* | cortex | Dali Co., Yunnan | June | HPLC | 37.8* | Feng et al., 2012 |
| *P. ludlowii* | phloem | Dali Co., Yunnan | June | HPLC | 22.2* | Feng et al., 2012 |
| *P. ludlowii* | xylem | Dali Co., Yunnan | June | HPLC | 18.0* | Feng et al., 2012 |
| *P. ludlowii* | stem | Dali Co., Yunnan | June | HPLC | 20.5* | Feng et al., 2012 |
| *P. ludlowii* | leaf | Dali Co., Yunnan | June | HPLC | 55.4* | Feng et al., 2012 |
| *P. ludlowii* | fruit | Dali Co., Yunnan | June | HPLC | 57.2* | Feng et al., 2012 |
| *Paeonia lactiflora* | cortex | Lianyungang Co., Jiangsu | August 20 (Before wilting) | HPLC | 48.9* | Wu and Zhao, 2005 |
| *P. lactiflora* | phloem | Lianyungang Co., Jiangsu | August 20 (Before wilting) | HPLC | 26.1* | Wu and Zhao, 2005 |
| *P. lactiflora* | xylem | Lianyungang Co., Jiangsu | August 20 (Before wilting) | HPLC | 33.3* | Wu and Zhao, 2005 |
| *P. lactiflora* | cortex | Lianyungang Co., Jiangsu | September 20 (After wilting) | HPLC | 44.2* | Wu and Zhao, 2005 |
| *P. lactiflora* | phloem | Lianyungang Co., Jiangsu | September 20 (After wilting) | HPLC | 27.7* | Wu and Zhao, 2005 |
| *P. lactiflora* | xylem | Lianyungang Co., Jiangsu | September 20 (After wilting) | HPLC | 24.8* | Wu and Zhao, 2005 |
| *P. lactiflora* | fresh flower | Weichang Co., Hebei | May | HPLC | Below limit | Jian et al., 2010 |
| *P. lactiflora* | green leaf | Weichang Co., Hebei | July | HPLC | 9.2* | Jian et al., 2010 |
| *P. lactiflora* | green stem | Weichang Co., Hebei | July | HPLC | 18.5* | Jian et al., 2010 |
| *P. lactiflora* | dry leaf | Weichang Co., Hebei | October | HPLC | Below limit | Jian et al., 2010 |
| *P. lactiflora* | dry stem | Weichang Co., Hebei | October | HPLC | 1.6* | Jian et al., 2010 |
| *P. lactiflora* | root | Weichang Co., Hebei | August | HPLC | 68.8* | Jian et al., 2010 |
| *P. lactiflora* | rhizomes | Weichang Co., Hebei | August | HPLC | 35.9* | Jian et al., 2010 |
| *P. lactiflora* | fruit | Weichang Co., Hebei | October | HPLC | Below limit | Jian et al., 2010 |
| *P. lactiflora* | seed | Weichang Co., Hebei | October | HPLC | 14.8* | Jian et al., 2010 |
| *P. lactiflora* | big root (>1cm) | Heihe Co., Heilongjiang | \ | HPLC | 56.1* | Hu et al., 2000 |
| *P. lactiflora* | medium root (0.5-1cm) | Heihe Co., Heilongjiang | \ | HPLC | 79.8* | Hu et al., 2000 |
| *P. lactiflora* | small root (<0.5cm) | Heihe Co., Heilongjiang | \ | HPLC | 45.7* | Hu et al., 2000 |
| *P. lactiflora* | rhizomes | Heihe Co., Heilongjiang | \ | HPLC | 42.7* | Hu et al., 2000 |
| *P. lactiflora* | root | Heihe Co., Heilongjiang | \ | HPLC | 39.9* | Hu et al., 2000 |
| *P. lactiflora* | root | Heihe Co., Heilongjiang | \ | HPLC | 53.6* | Hu et al., 2000 |
| *P. lactiflora* | rhizomes | Heihe Co., Heilongjiang | \ | HPLC | 92.1* | Hu et al., 2000 |
| *P. lactiflora* | big root (>1cm) | Duolun Co., Inner Mongolia | \ | HPLC | 55.1* | Hu et al., 2000 |
| *P. lactiflora* | medium root (0.5-1cm) | Duolun Co., Inner Mongolia | \ | HPLC | 77.7* | Hu et al., 2000 |
| *P. lactiflora* | small root (<0.5cm) | Duolun Co., Inner Mongolia | \ | HPLC | 63.4* | Hu et al., 2000 |
| *P. lactiflora* | rhizomes | Duolun Co., Inner Mongolia | \ | HPLC | 50.3* | Hu et al., 2000 |
| *P. lactiflora* | big root (>1cm) | Weichang Co., Hebei | \ | HPLC | 39.1* | Hu et al., 2000 |
| *P. lactiflora* | medium root (0.5-1cm) | Weichang Co., Hebei | \ | HPLC | 52.9* | Hu et al., 2000 |
| *P. lactiflora* | small root (<0.5cm) | Weichang Co., Hebei | \ | HPLC | 62.4* | Hu et al., 2000 |
| *P. lactiflora* | rhizomes | Weichang Co., Hebei | \ | HPLC | 77.1* | Hu et al., 2000 |
| *P. lactiflora* | root | Weichang Co., Hebei | \ | HPLC | 68.2* | Hu et al., 2000 |
| *P. lactiflora* | root | Huanglong Co., Shaanxi | \ | HPLC | 55.2* | Hu et al., 2000 |
| *P. lactiflora* | rhizomes | Huanglong Co., Shaanxi | \ | HPLC | 63.9* | Hu et al., 2000 |
| *P. lactiflora* | leaf | Beijing | \ | HPLC | 3.8* | Hu et al., 2000 |
| *P. lactiflora* | stem | Beijing | \ | HPLC | 1.5* | Hu et al., 2000 |
| *P. lactiflora* | rhizomes | Beijing | \ | HPLC | 41.5* | Hu et al., 2000 |
| *P. lactiflora* | root | Beijing | \ | HPLC | 24.5* | Hu et al., 2000 |
| *P. lactiflora* | leaf | Ziyang Co., Shaanxi | \ | HPLC | 14.3* | Hu et al., 2000 |
| *P. lactiflora* | stem | Ziyang Co., Shaanxi | \ | HPLC | 3.3* | Hu et al., 2000 |
| *P. lactiflora* | rhizomes | Ziyang Co., Shaanxi | \ | HPLC | 36.0* | Hu et al., 2000 |
| *P. lactiflora* | root | Ziyang Co., Shaanxi | \ | HPLC | 28.7* | Hu et al., 2000 |
| *P. lactiflora* | main root | Jinyun Co, Zhejiang | \ | HPLC | 32.1* | Hu et al., 2000 |
| *P. lactiflora* | branch root | Jinyun Co, Zhejiang | \ | HPLC | 28.7* | Hu et al., 2000 |
| *P. lactiflora* | root | Jinyun Co, Zhejiang | \ | HPLC | 17.0* | Hu et al., 2000 |
| *P. lactiflora* | main root | Dongyang Co, Zhejiang | \ | HPLC | 20.7* | Hu et al., 2000 |
| *P. lactiflora* | branch root | Dongyang Co, Zhejiang | \ | HPLC | 28.4* | Hu et al., 2000 |
| *P. lactiflora* | fibrous root | Dongyang Co, Zhejiang | \ | HPLC | 26.9* | Hu et al., 2000 |

*The content in the original study was expressed as the percentage.
